# Lung perfusion assessed by SPECT/CT after a minimum of three months anticoagulation therapy in patients with SARS-CoV-2-associated acute pulmonary embolism: a retrospective observational study

**DOI:** 10.1186/s12931-022-02188-2

**Published:** 2022-10-31

**Authors:** Thomas M. Berghaus, Stefanie Bader, Christian Faul, Sabine Haberl, Florian Schwarz, Alessandro Liebich, Alexander Dierks, Malte Kircher, Constantin Lapa, Christian H. Pfob

**Affiliations:** 1 Department of Cardiology, Respiratory Medicine and Intensive Care, University Hospital Augsburg, University of Augsburg, Stenglinstrasse 2, D-86156 Augsburg, Germany; 2 Nuclear Medicine, Faculty of Medicine, University of Augsburg, Augsburg, Germany; 3 Department of Radiology, University Hospital Augsburg, University of Augsburg, Augsburg, Germany; 4 Ludwig-Maximilians-University Munich, Munich, Germany

**Keywords:** Acute pulmonary embolism, Anticoagulation therapy, COVID-19, DOAC, Persistent pulmonary perfusion defects, SARS-CoV-2, SPECT/CT

## Abstract

**Background:**

Anticoagulant treatment is recommended for at least three months after severe acute respiratory syndrome coronavirus 2 (SARS-CoV-2)-related acute pulmonary embolism (PE), but the persistent pulmonary clot burden after that time is unknown.

**Methods:**

Lung perfusion was assessed by ventilation-perfusion (V/Q) SPECT/CT in 20 consecutive patients with SARS-CoV-2-associated acute PE after a minimum of three months anticoagulation therapy in a retrospective observational study.

**Results:**

Remaining perfusion defects after a median treatment period of six months were observed in only two patients. All patients (13 men, seven women, mean age 55.6 ± 14.5 years) were on non-vitamin K direct oral anticoagulants (DOACs). No recurrent venous thromboembolism or anticoagulant-related bleeding complications were observed. Among patients with partial clinical recovery, high-risk PE and persistent pulmonary infiltrates were significantly more frequent (p < 0.001, respectively).

**Interpretation:**

Temporary DOAC treatment seems to be safe and efficacious for resolving pulmonary clot burden in SARS-CoV-2-associated acute PE. Partial clinical recovery is more likely caused by prolonged SARS-CoV-2-related parenchymal lung damage rather than by persistent pulmonary perfusion defects.

## Introduction

The coronavirus disease 2019 (COVID-19), caused by the severe acute respiratory syndrome coronavirus 2 (SARS-CoV-2), is a global infectious disease with an enormous impact on public health. The SARS-CoV-2 infection primarily affects the respiratory system, but it may involve different organ systems and is associated with an increased risk for significant coagulopathy, resulting in high rates of venous thromboembolism (VTE), especially in severely ill patients.

There is limited evidence as to how long patients with SARS-CoV-2-related acute pulmonary embolism (PE) should be treated in order to restore pulmonary arterial blood flow. At present, standard VTE management-conform non-COVID recommendations are generally advised, recommending a treatment with anticoagulants for at least three months [1]. However, little is known about the persistent pulmonary clot burden after that time [2], and there is even emerging evidence that the thrombotic risk might persist after recovery from COVID-19 [3, 4].

We therefore evaluated lung perfusion assessed by ventilation-perfusion (V/Q) single photon emission computed tomography/computed tomography (SPECT/CT) after a minimum of three months anticoagulation therapy in patients with SARS-CoV-2-associated acute PE in order to determine the optimal treatment regime.

## Methods

All patients who were diagnosed with SARS-CoV-2-associated acute PE between 1. April 2020 and 31. April 2021 at the University Hospital Augsburg were retrospectively identified. They underwent V/Q SPECT/CT imaging for the re-evaluation of persistent perfusion defects after a minimum period of three months sufficient anticoagulation treatment, unless they had an alternative diagnosis for ongoing anticoagulation therapy (e.g. atrial fibrillation, active cancer, recurrent VTE, known hypercoagulatory state, etc.). First, ventilation with an 99 m-technetium- (99 m-Tc-) labelled aerosol (Technegas®;Curium Pulmotec®, Ulm, Germany) produced by a nebulizer (Technegas™ Generator, Cyclomedica Australia Pty Ltd., Kingsgrove, Australia) was performed followed by SPECT/CT imaging using a dual-head SPECT/CT gamma camera (GE Optima™ NM/CT 670 or Siemens Symbia T2). Low-dose CT for attenuation correction was acquired in respiratory center position with ventilation SPECT/CT scanning. Iterative and attenuation corrected images were reconstructed using ordered-subset expectation maximization. Subsequently, an activity of about 150 MBq 99 m-Tc-Pulmocis® (Curium Pulmocis) was injected intravenously and SPECT scanning was performed again, using the same imaging parameters as described above. Images were reviewed by two board certified nuclear medicine physicians and diagnosis was reached in consensus.

All patients gave written informed consent for lung perfusion imaging. The Ethics Committee at the Ludwig-Maximilians-University Munich, Germany exempted this retrospective, non-interventional observational study with a waiver of patient informed consent on May 20, 2021.

The statistical analysis was performed using IBM SPSS® Statistics version 25. Continuous variables were given as mean values with standard deviations. Differences in continuous variables were analysed by Student’s t-test. Categorical variables were specified as amounts (with percentages of total) and their distribution was further analysed by the chi-square test or by Fisher’s test in case of low frequencies. A p-value of less than 0.05 was considered to indicate statistical significance.

## Results

Between 1. April 2020 and 31. April 2021, 35 consecutive patients with SARS-CoV-2-associated acute PE were identified. Out of these patients, three patients died during hospitalisation and eight patients had an alternative diagnosis for ongoing anticoagulation therapy. From the 24 eligible patients, three patients were lost for follow-up due to geographical reasons and one patient denied lung perfusion imaging. There were no deaths observed during the follow-up period. Thus, 20 patients could be included in the retrospective study.

Patients’ characteristics are summarised in Table 1. In most patients, V/Q SPECT/CT was performed six months (median six months, range three – ten months) after SARS-CoV-2-associated acute PE. All patients were on DOAC therapy during the entire follow-up period. No VTE recurrence or anticoagulant-related bleeding complications were observed. Within the study population (13 men, seven women, mean age 55.6 ± 14.5 years), only two patients (10.0%) showed remaining perfusion defects on V/Q SPECT/CT (both female, aged 71 and 87 years, one with full, the other with partial clinical recovery) six months after SARS-CoV-2-associated acute PE. In seven out of 20 patients (35.0%), clinical recovery was partial. When comparing patients with full and those with only partial recovery, persistent pulmonary infiltrates were completely absent in fully recovered patients (p < 0.001). Among patients with only partial clinical recovery, high-risk PE was significantly more frequent (p < 0.001). There were no statistically significant differences in gender, age, pulmonary thrombus load or remaining perfusion defects between the two groups (Table 2).

**Table 1:** Clinical parameters of patients with SPECT/CT-follow up after COVID-19 associated PE

			COVID-19 disease							SPECT/CT-follow up							
**№**	**Gender**	**Age**	**Respiratory support**	**Specific COVID therapy**	**COVID rehab**	**Severity of PE** [13]	**Thrombolysis**	**Pulmonary thrombus localization**	**Quanadli-** **score**	**Remaining perfusion defects**	**New perfusion defects**	**Persistent pulmonary infiltrates**	**Duration of OAC treatment**	**Type of OAC**	**OAC-related** **bleeding**	**VTE recurrence**	**Clinical recovery**
1	male	33	high-flow oxygen	steroid, convalescent plasma	no	low-risk	no	segmental	15	no	no	no	6 months	DOAC	no	no	full
2	male	33	ECMO	steroid, convalescent plasma	no	low-risk	no	segmental	3	no	no	no	3 months	DOAC	no	no	full
3	male	57	oxygen	steroid	yes	low-risk	no	segmental	9	no	no	no	6 months	DOAC	no	no	partial
4	male	54	NIV	steroid, convalescent plasma	no	high-risk	yes	central	32	no	no	yes	6 months	DOAC	no	no	partial
5	male	61	IV	steroid, convalescent plasma	yes	low-risk	no	segmental	8	no	no	yes	10 months	DOAC	no	no	partial
6	male	42	oxygen	vitamin D	no	low-risk	no	segmental	1	no	no	no	6 months	DOAC	no	no	full
7	female	50	oxygen	no	no	low-risk	no	segmental	13	no	no	no	6 months	DOAC	no	no	full
8	female	77	NIV	steroid, convalescent plasma	yes	high-risk	yes	segmental	9	no	no	yes	6 months	DOAC	no	no	partial
9	male	79	NIV	steroid, convalescent plasma	no	intermediate-low-risk	no	segmental	16	no	no	no	6 months	DOAC	no	no	full
10	female	87	oxygen	vitamin D	no	low-risk	no	segmental	1	yes	no	yes	6 months	DOAC	no	no	partial
11	female	36	oxygen	no	no	intermediate-low-risk	no	segmental	1	no	no	no	6 months	DOAC	no	no	full
12	male	62	oxygen	no	no	low-risk	no	segmental	2	no	no	no	6 months	DOAC	no	no	full
13	female	56	none	no	no	low-risk	no	segmental	-	no	no	no	6 months	DOAC	no	no	full
14	male	46	oxygen	steroid	no	intermediate-low-risk	no	segmental	5	no	no	no	6 months	DOAC	no	no	full
15	male	47	oxygen	no	no	low-risk	no	segmental	-	no	no	yes	3 months	DOAC	no	no	partial
16	female	71	oxygen	no	no	intermediate-high-risk	no	central	17	yes	no	no	6 months	DOAC	no	no	full
17	male	63	NIV	convalescent plasma	yes	low-risk	no	segmental	-	no	no	no	10 months	DOAC	no	no	full
18	female	55	oxygen	no	no	intermediate-low-risk	no	segmental	27	no	no	no	6 months	DOAC	no	no	partial
19	male	49	oxygen	steroid	no	intermediate-low-risk	no	segmental	8	no	no	no	4 months	DOAC	no	no	full
20	male	54	High-flow oxygen	remdesivir, steroid	no	Intermediate-high-risk	no	segmental	12	no	no	no	3 months	DOAC	no	no	full

**Table 2:** Differences in clinical parameters between subgroups with partial and full recovery

Clinical recovery	Partial n = 7	Full n = 13	P
Female gender [n (%)]	3 (42.9)	4 (30.8)	NS
Age [years]	62.6 ± 13.2	51.8 ± 13.7	NS
High-risk PE [n (%)]	2 (28.6)	0	< 0.001
Quanadli-Score [points]	14.3 ± 11.2	8.1 ± 6.6	NS
Remaining perfusion defects [n (%)]	1 (14.3)	1 (7.7)	NS
Persistent pulmonary infiltrates [n (%)]	5 (71.4)	0	< 0.001

## Discussion

To the best of our knowledge, the present study is one of the first to evaluate lung perfusion after recovery from SARS-CoV-2-related acute PE. We found persistent pulmonary perfusion defects in 10% of study participants after six months of sufficient oral anticoagulant therapy. However, persistent pulmonary thrombus load does not seem to be associated with an unfavourable clinical short-term outcome.

Current recommendations suggest a temporary treatment with anticoagulants for at least three months in VTE patients with COVID-19, as SARS-CoV-2 is considered a relevant, transient provoking factor for VTE [5, 6]. SARS-CoV-2 provokes an inflammatory response with a release of cytokines, chemokines, and cell activation. Through interactions between inflammation, complement activation, and coagulation, a hypercoagulable state is generated [6]. General risk factors for VTE, such as older age, obesity, immobilisation, chronic kidney disease or malignancy are also relevant in COVID-19 patients. Hypoxia and sepsis in particular are common risk factors for VTE in SARS-CoV-2 infections [6]. As we showed that lung perfusion was restored in most patients after six months of sufficient oral anticoagulant therapy, our data support the notion that a temporary anticoagulant treatment might be sufficient for resolving pulmonary clot burden.

All patients were set on a DOAC therapy after recovery from COVID-19 in order to prevent VTE recurrence. As DOACs interact with a variety of COVID-19 drugs (resulting in a modified action, either enhancement or reduction, thus exposing patients to a potential risk of bleeding or thrombosis), DOACs are considered not to be a preferable therapy option during SARS-CoV-2 infections [7]. Therefore, all our patients were treated with low-molecular-weight or unfractionated heparin during hospitalisation and were switched to DOACs shortly before hospital discharge. In the outpatient setting, DOACs do have many advantages compared with vitamin K antagonists, as they are safer and do not require routine monitoring. As neither VTE recurrence nor anticoagulant-related bleeding complications were observed in our cohort, this study provides important real-life data suggesting that a DOAC therapy can be safe and efficacious in preventing PE recurrence after recovery from COVID-19.

In nearly 40% of our study population, clinical recovery was partial, and persistent pulmonary infiltrates were significantly more frequent in this subgroup. Pulmonary lesions at time of discharge from hospital were found to be quite common even in mild and moderate SARS-CoV-2 infections [8], but studies on radiographic abnormalities post-COVID-19 are sparse, especially in asymptomatic patients. Rates of persistent pulmonary lesions on CT scans at three-month follow-up have been estimated at between 40 and 70% in three earlier studies [9–11]. In contrast to these earlier reports, the percentage of persistent chest lesions in our study cohort is lower; however, our cohort was evaluated after a median period of six, not of three months, and lung infiltrates in some patients might have resolved meanwhile.

In contrast to one study, which found that chest radiography is a poor predictor of clinical and functional impairment eight weeks after severe COVID-19 pneumonia [12], other reports clearly depict a significant and strong correlation between abnormalities on chest radiography or CT and functional capacity 12 weeks after COVID-19-related hospitalisations [9, 11]. This correlation might even be stronger after six months of follow-up. We therefore conclude that partial clinical recovery in our study cohort is more likely caused by prolonged SARS-CoV-2-related parenchymal lung damage than by persistent pulmonary perfusion defects.

We admit that our study is limited by the single-centre design and its very small sample size. Therefore, our data are preliminary and need to be confirmed in a larger study population. However, the demographic profile of our cohort seems to be very representative for a general COVID-19 patient population. Unfortunately, regular lung function tests were not performed during follow-up in our trial. Further limitations are the retrospective study design and the short-term observational period.

Despite these limitations, we conclude that a temporary DOAC treatment might be sufficient for resolving pulmonary clot burden after SARS-CoV-2-associated acute PE. In addition, partial clinical recovery seems to be more likely caused by prolonged COVID-19-related parenchymal lung damage than by persistent pulmonary perfusion defects.

## Data Availability

Data are available upon reasonable request from the corresponding author.

## Abbreviations

COVID-19: coronavirus disease 2019
CTPA: computed tomographic pulmonary angiography
DOAC: non-vitamin K direct oral anticoagulants
ICU: intensive care unit
PE: pulmonary embolism
SARS-CoV-2: severe acute respiratory syndrome coronavirus 2
(V/Q) SPECT/CT: ventilation-perfusion single photon emission computed tomography/computed tomography
VTE: venous thromboembolism
